# The hypervascularization of the nail matrix and nail bed as a predictor of nail psoriasis

**DOI:** 10.1002/ski2.85

**Published:** 2021-12-24

**Authors:** D. Becker‐Capeller, S. El‐Nawab‐Becker, M. Töllner, A. Kleinheinz, T. Witte

**Affiliations:** ^1^ Rheumatologic Practice Clinic Dr. Hancken Stade Germany; ^2^ Elbe Clinic, Department of Dermatology, Buxtehude Germany; ^3^ Rheumatology and Immunology Medical School Hannover Hannover Germany

## AUTHOR CONTRIBUTIONS

**Detlef Becker‐Capeller:** Conceptualization, Data curation, Investigation, Project administration, Resources, Software, Supervision, Writing – original draft, Writing – review & editing. **Soham El‐Nawab‐Becker:** Conceptualization, Data curation, Formal analysis, Investigation, Resources, Writing – review & editing. **Malo Toellner:** Data curation, Formal analysis, Investigation, Software, Validation, Writing – original draft, Writing – review & editing. **Andreas Kleinheinz:** Data curation, Investigation, Supervision. **Torsten Witte:** Formal analysis, Methodology, Project administration, Supervision, Validation, Visualization, Writing – review & editing.

## ETHICS STATEMENT

This study was approved by the Ethics Committee of the MHH, Medical School Hannover, Germany (7734_BO‐S_2018) and conducted in accordance with the EU General Data Protection Regulation (GDPR) and applicable local regulations. Patient confidentiality was maintained according to local laws, and informed consent was obtained for publication.

Dear Editor,

1

Nail psoriasis (NailPsO) is an extreme diagnostic and therapeutic challenge. NailPso is the strongest predictor of psoriatic arthritis (PsA) and a serious stigma for the patients.^1^, ^2^ The synovio‐entheseal concept^3^ has helped us establish an anatomical‐pathophysiological relationship between the DIP joint, extensor tendon, and nail matrix. In our day‐to‐day practice, we have observed that hypervascularization (HV) in ultrasound Power Doppler (US‐PD) of the nail matrix may be a pathognomonic element of nail PsO on its own. However, it remains unclear whether there is a difference in the ultrasound PD examination of the DIP joint and nail area in patients with psoriasis vulgaris and nail psoriasis versus patients with psoriasis vulgaris without nail psoriasis In this monocentric prospective study, all consecutive patients with freshly diagnosed psoriasis (<2 years) who were referred by a dermatologist to a rheumatology practice for clarification of PsA were included. Dermatologic diagnoses such as lichen ruber or eczema were excluded dermatologically in advance. In addition to demographic data, assessments (PASI, DLQI, CASPAR, GEPARD, DAS28, SJ, TJ, FFBH), and clinical examination, we performed a standardized ultrasound PD examination of the affected fingertips and the corresponding DIP joints in PsO patients suffering from nail psoriasis. The diagnosis of nail psoriasis was made clinically when oil‐drop phenomenon, onycholysis, subungual hyperkeratosis, and nail pittings (>5) were detectable. We also performed corresponding examinations of the right second and third finger in PsO patients without nail involvement. We enroled 69 patients during the study period (12 months, 2019–2020). Of these, 25 PsO patients were without nail involvement, and 44 PsO patients had nail involvement. There was no difference in age, BMI, and sex in both groups (PsO and NailPso). Positive Power Doppler hypervascularization score (PD‐HV score) of 2–3, a definite sign of activity,^4^, ^5^ was seen at the extensor tendon in 59.0% of patients with nail psoriasis and 62.1% of patients without nail psoriasis. This suggests that extensor tendon inflammation occurs as often in nail psoriasis patients as in patients without nail psoriasis. At the nail matrix, hypervascularization was seen in 92.3% of patients with nail psoriasis (36 pat.) with a PD HV score of 2–3. In 7.7% of the patients with nail psoriasis, no hypervascularization could be detected. In 10 patients without nail psoriasis (34.5%), an HV score of 2–3 was seen. In 19 patients (65.5%) without nail PsO, no hypervascularization was seen. This demonstrated a clear correlation between nail psoriasis and inflammatory signs of the nail matrix. The two patient groups differed significantly in terms of the frequency of inflammatory change of the nail matrix and nail bed (*p* < 0.001) (Table 1).

**TABLE 1:** US‐powerdoppler (US‐PD) Hypervascularisation (HV) score

	Score	All patients (%)	Patients without NailPso (%)	Patients with NailPso (%)	*p*‐value^a^
Ext. Tendon HV score	0/1	27 (39.7)	11 (37.9)	16 (41.0)	
	2/3	41 (60.3)	18 (62.1)	23 (59.0)	1.000
Nail matrix HV score	0/1	22 (32.4)	19 (65.5)	3 (7.7)	
	2/3	46 (67.6)	10 (34.5)	36 (92.3)	<0.001
Nail bed HV score	0/1	46 (67.6)	27 (93.1)	19 (48.7)	
	2/3	22 (32.4)	2 (6.9)	20 (51.3)	<0.001

^a^ Exact Fisher‐test.

The best of our knowledge, this is the first study to show that hypervascularization of the nail matrix as a sign of inflammation in psoriasis patients with nail psoriasis was significantly common than in patients without nail psoriasis. US‐PD examination is a simple and non‐invasive procedure that can be performed routinely in daily practice. The hypervascularization of the nail matrix should prompt clinicians to suspect nail psoriasis in the early stage of PsO.^6^ The results should be reviewed in future studies also with regard to dermatological differential diagnoses.

## CONFLICT OF INTEREST

None to declare.

## Data Availability

Data are available on individual request by the corresponding author.
